# Case Report: Pediatric Erdheim-Chester disease with bilateral chorioretinal and central nervous system infiltration

**DOI:** 10.3389/fmed.2026.1796505

**Published:** 2026-04-01

**Authors:** Hongyu Zhong, Licong Liang, Fang Lu

**Affiliations:** Department of Ophthalmology, West China Hospital, Sichuan University, Chengdu, Sichuan, China

**Keywords:** chorioretinal infiltration, central nervous system infiltration, bilateral chorioretinal and central nervous system infiltration, Erdheim-Chester disease, pediatric Erdheim-Chester disease

## Abstract

Erdheim-Chester disease (ECD) is a rare histiocytic disorder characterized by the infiltration of tissues with foamy, xanthomatous CD68/CD163-positive, CD1a-negative histiocytosis. Pediatric ECD with chorioretinal involvement is exceedingly rare. Clinical manifestations and ophthalmic imaging findings are atypical, leading to misdiagnosis or missed diagnosis. From an ophthalmological perspective, we share an extremely rare case of pediatric ECD with chorioretinal and central nervous system infiltration, highlighting the diagnostic challenges and expanding the phenotypic spectrum of intraocular manifestations.

## 1 Introduction

Erdheim-Chester disease (ECD) is an extremely rare non-Langerhans-cell histiocytosis, with only approximately 1,500 documented cases worldwide to date (1). ECD predominantly affects males (3:1) between 40 and 70 years of age, with a mean diagnostic age of approximately 55 years (2). Pediatric onset is exceptional, with less than 20 cases to date (3). ECD is characterized by the infiltration of tissues with foamy, xanthomatous CD68/CD163-positive, CD1a-negative histiocytoses. The *BRAF (V600E)* variant—present in approximately 51 to 54% of cases, has both diagnostic and therapeutic relevance because of its relative rarity in other histiocytosis (4).

The initial clinical manifestations can vary, typically later progressing to sequential multisystem involvement (5). Ophthalmic involvement occurs in nearly one-third of patients (6). Palpebral and periocular xanthelasma are the most common clinical manifestation, whereas orbital infiltration is reported in approximately one quarter of cases with symptoms related to a space-occupying lesion. Intraocular infiltration is considered extremely rare, and when it occurs it generally involves the choroid (7). Cases of pediatric choroidal involvement in ECD are exceedingly rare. To date, no pediatric ECD cases with confirmed intraocular involvement have been documented in the published literature (8).

From an ophthalmological perspective, we report a pediatric case of ECD presenting with bilateral choroidal and central nervous system (CNS) involvement. This case highlights the diagnostic challenges and expands the phenotypic spectrum of intraocular manifestations in pediatric ECD.

## 2 Case report

A 6-year-old boy was referred to West China Hospital for left-eye choroidal masses incidentally found after ocular trauma. He was previously healthy, with no history of prematurity, oxygen supplementation or inherited disorders.

**Figure 1:**
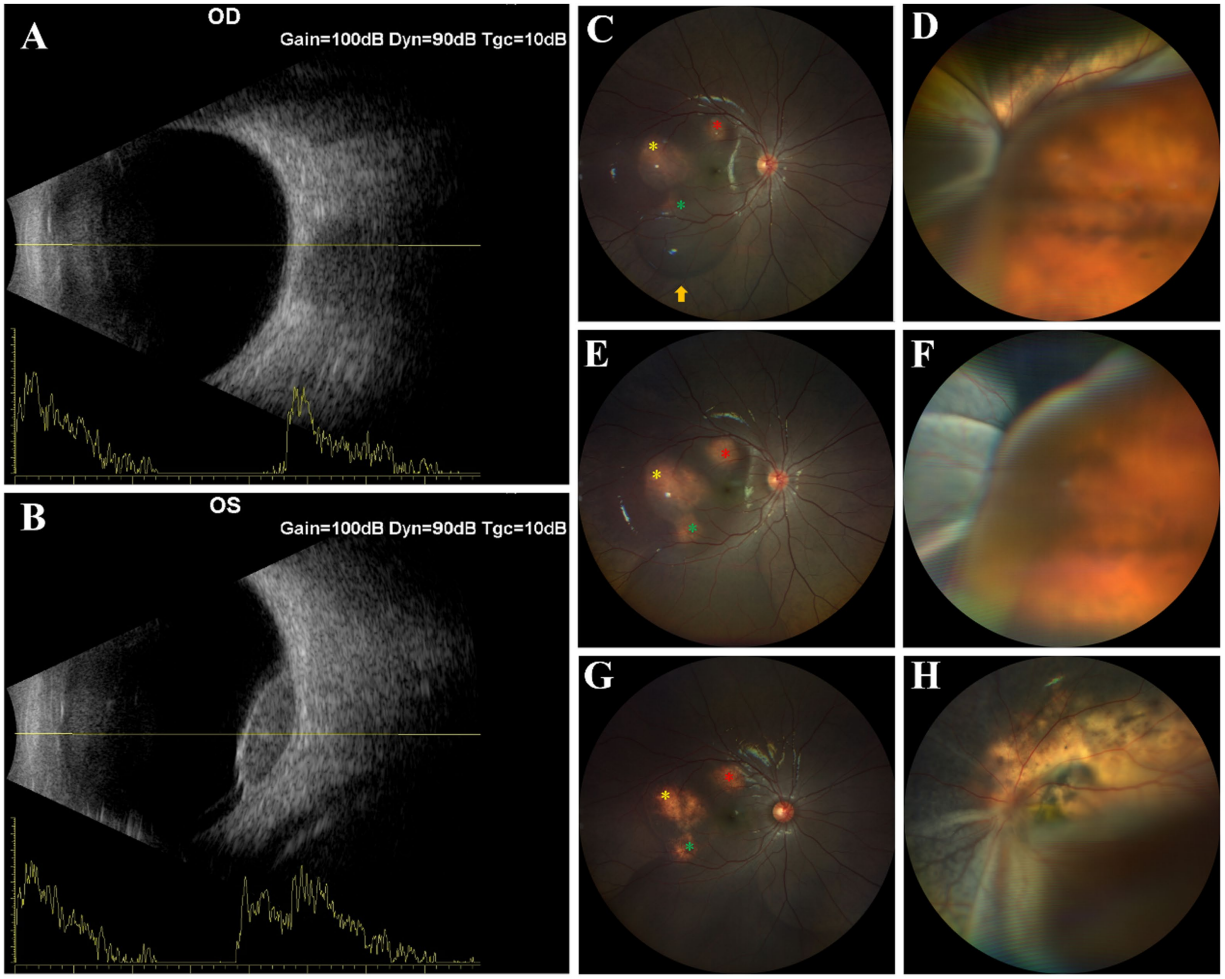
*First follow-up examination* **(A,B)**: B-scan ultrasound demonstrated a solid mass-like echogenic lesion with well-defined borders and homogeneous internal echogenicity in the posterior vitreous in left eye. A linear adherent echo connecting the lesion to the ocular wall is noted. Suspicious for choroidal neoplastic lesion with retinal detachment. Right eye reveals no significant abnormal echoes in the mid-posterior vitreous. *Second follow-up examination* **(C,D)**: Fundus examination revealed three orange-yellow circular choroidal lesions (red, yellow, and blue asterisks) with associated subretinal fluid in the right eye. Gravity-dependent accumulation of exudative fluid inferior to the lesions resulted in localized retinal detachment (yellow arrow). In the left eye, a large area of yellowish-white choroidal lesions was presented, accompanied by extensive retinal detachment involving the macula. *Third follow-up examination* **(E,F)**: Fundus examination revealed progressive enlargement of choroidal lesions in the right eye, complicated by an expanded area of inferior retinal detachment. The left eye exhibited discernible progression in the retinal detachment region. *Fourth follow-up examination* **(G,H)**: Fundus examination revealed reduced choroidal lesions, resolved SRF with reattachment and pigmentation in right eye and improved RD, large yellowish-white choroidal lesions with perilesional pigmentation, posterior pole subretinal fibrosis, and attenuated or obliterated retinal arteries in left eye.

At baseline, best corrected visual acuity (BCVA) was 20/33 (right eye) and 20/40 (left eye); intraocular pressure (IOP) was normal bilaterally. Slit lamp examination was unremarkable. B-scan ultrasonography revealed a solid choroidal mass with serous RD in the left eye, while the right eye was normal (Figures 1A,B). The patient underwent two sessions of photodynamic therapy (PDT) and plaque radiotherapy (PRT) for suspected choroidal hemangioma at another hospital, with no visual improvement and left eye exotropia subsequently developed.

The patient returned for follow-up. BCVA was 20/25 (right eye) and light perception (left eye). Ocular examination showed left exotropia and normal bilateral anterior segments. Fundus examination revealed three orange-yellow circular choroidal lesions with subretinal fluid (SRF) in the right eye; gravity-dependent exudative fluid accumulation inferior to the lesions caused localized retinal detachment (RD). The left eye had a large yellowish-white choroidal lesion with extensive macular-involving RD (Figures 1C,D).

**Figure 2:**
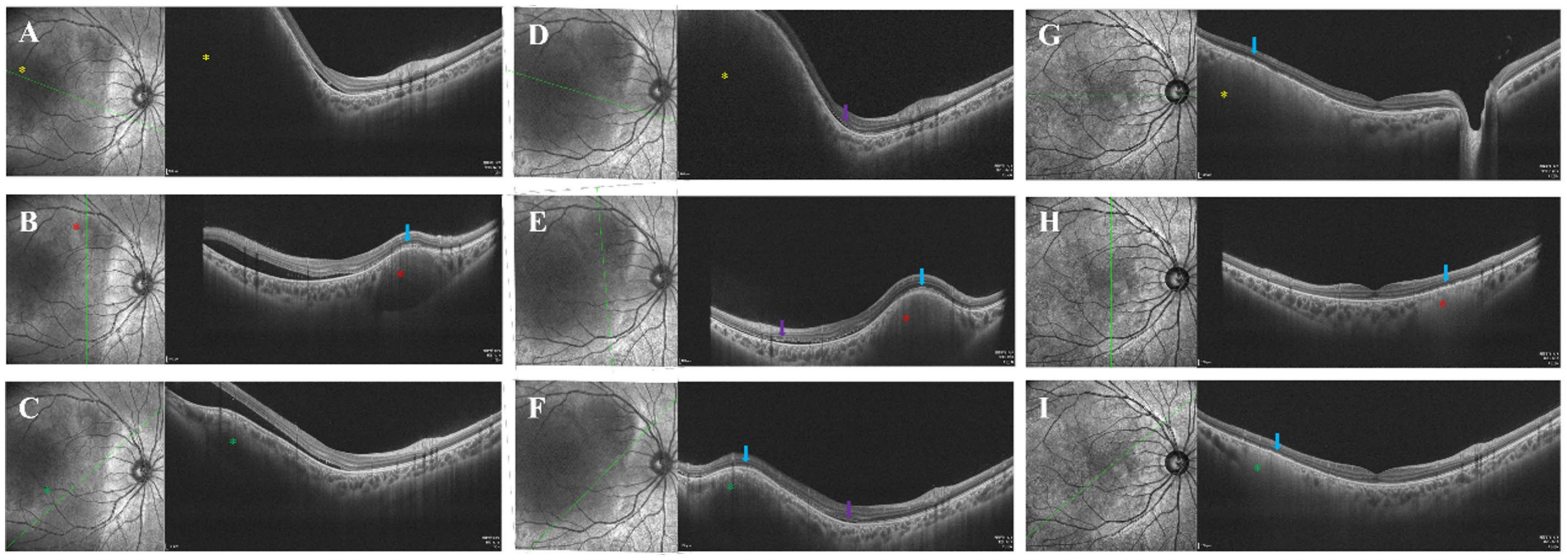
*Second follow-up examination* **(A–C)**: SS-OCT demonstrated hyporefletive choroidal lesion, hyperreflective exudative material between the inner retina and retinal pigment epithelium corresponding to the choroidal lesions, with associated SRF surrounding the lesion periphery in the right eye. The right eye’s choroidal lesions showed complete disruption of the ellipsoid zone and obliteration of Haller’s layer, accompanied by subfoveal choroidal thickening. *Third follow-up examination* **(D–F)**: In addition to the aforementioned changes, the right eye exhibited shark-tooth-like alterations extending from the outer plexiform layer to the outer nuclear layer (purple arrow), and all three choroidal lesions have demonstrated progressive enlargement. *Fourth follow-up examination* **(G–I)**: SS-OCT revealed that the SRF had completely resolved, and the retina was reattached in the right eye. However, hyperreflective material persisted in the outer retinal layers corresponding to the choroidal lesion, with significant improvement noted in choroidal lesion. Due to severe retinal detachment in the patient’s left eye before chemotherapy, the OCT scan could not be performed. The red, yellow, and green asterisks in SS-OCT correspond to those of the same color in fundus photography.

Swept-source optical coherence tomography (SS-OCT) confirmed choroidal mass elevation in the right eye consistent with fundus findings, showing hyper-reflective exudative material (HREM) between the neurosensory retina and retinal pigment epithelium (RPE), along with ellipsoid zone disruption, perilesional SRF, Haller’s layer obliteration, and subfoveal choroidal thickening (Figures 2A–C). SS-OCT was unavailable for the left eye due to extensive RD. Two weeks later, follow-up showed marked enlargement of choroidal lesions and bilateral RD progression (Figures 1E,F, 2E–G). Whole exome sequencing identifies no pathogenic variants.

**Figure 3:**
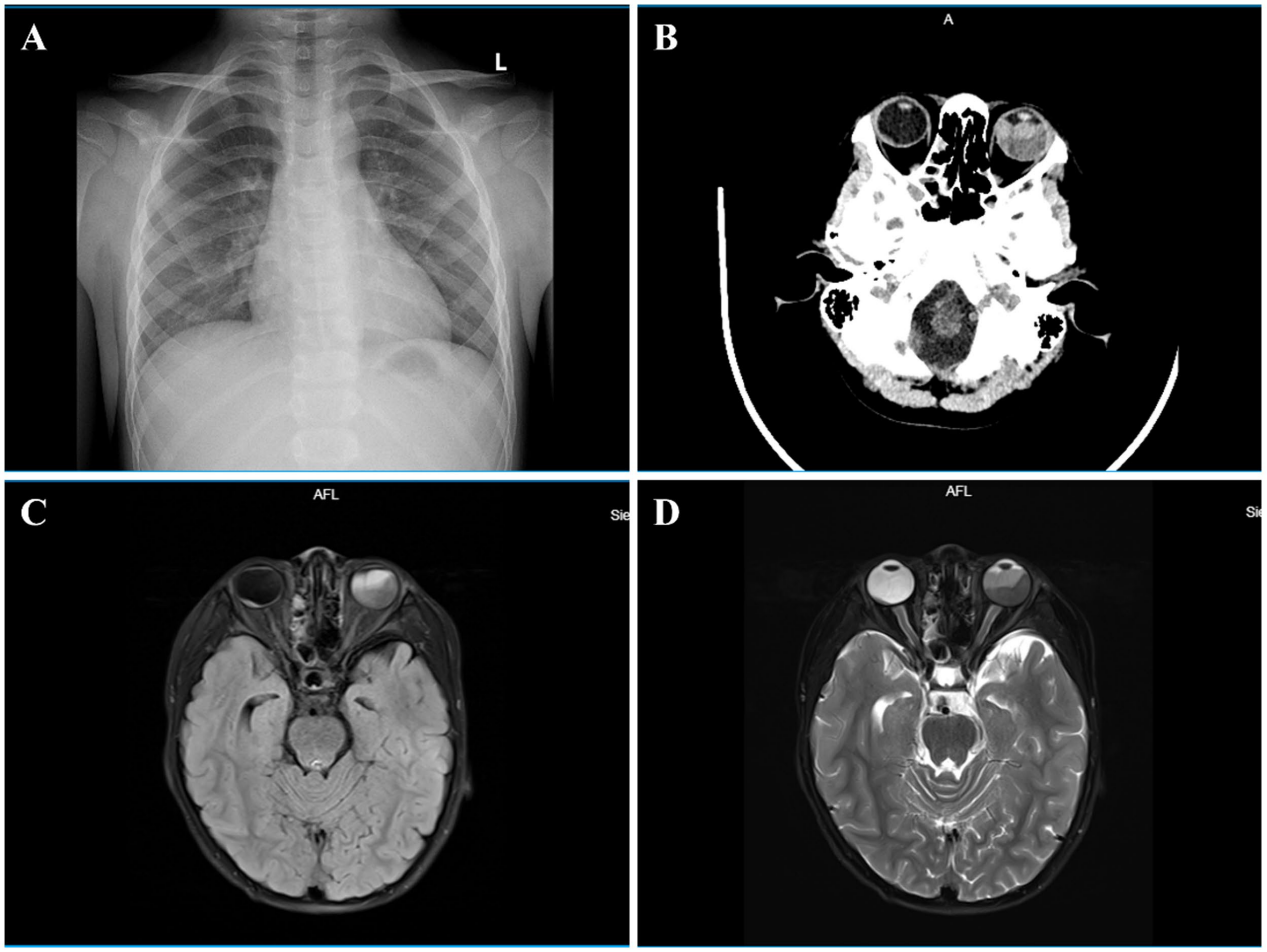
*Chest X-ray* (anteroposterior view) revealed no cardiopulmonary abnormalities in the pediatric patient **(A)**. *Cranial CT* showed a heterogeneous-density lesion (maximal cross-section 1.8 × 2.7 cm) within the left eyeball with inhomogeneous contrast enhancement and mild morphological distortion, suggestive of neoplastic lesions **(B)**. *Cranial MRI* further revealed morphological distortion of the left eyeball containing a nodular mixed-signal lesion (maximal transverse section 2.3 × 1.8 cm), exhibiting slightly hyperintense signal on T1WI, layered hypointense to mildly hypointense signal on T2WI, corresponding to hypointense or slightly hyperintense signal on FLAIR, with post-contrast heterogeneous enhancement in T2WI-hypointense areas and no significant enhancement in T2WI-mildly hypointense regions. Additionally, mild thickening and enhancement were noted in the posterior wall of the right eyeball **(C,D)**.

Based on clinical and imaging findings, choroidal melanoma and hemangioma were ruled out. Multimodal imaging (cranial CT, MRI, PET-CT) provided diagnostic clues (Figure 3): CT showed a mixed-density, heterogeneously enhancing neoplastic lesion in left eye and multiple intracranial soft tissue masses; MRI revealed a mixed-signal nodule in left eye (T1-hyperintense, T2-hypointense with laminar appearance, heterogeneous enhancement in T2-hypointense areas), mild posterior segment thickening and enhancement in the right eye, and multiple significantly enhancing isointense T1/T2 intracranial nodules; PET-CT demonstrated FDG-avid, mildly hyperdense intracranial nodules and an FDG-elevated hyperdense nodule in the left posterior eye.

The patient underwent right ventricular space-occupying lesion resection. Histopathology showed atypical cellular proliferation with marked eosinophilic infiltration (suspicious for neoplasia). Immunohistochemistry: S100 (+), CD1a (focal+), Langerin (focal+), CD163 (+), Cyclin D1 (+), Oct2 (+), PU.1 (+), CD68 (+), KI-67 (+, ~15%), SOX10 (−), GFAP (−), ALK (−), ROS1 (−). Real-time fluorescent quantitative PCR detected a BRAF (V600E) variant (T > A substitution at nucleotide 1799). External pathological review showed diffuse histiocytic, focal chronic inflammatory and diffuse eosinophilic infiltration, without Langerhans cell proliferation. Immunohistochemistry: CD163 (+), CD68 (+), Lysozyme (+), Fascin (+), F13a (+), S-100 (+), Ki-67 (10%); CD1a (−), Langerin (−), BRAF (V600E) (−), CD23 (−). Integrating clinical course, multimodal imaging, histopathology and molecular findings, the patient was diagnosed with ECD. He received inpatient cyclic chemotherapy (intravenous cytarabine 100 mg/day + dexamethasone 3.2 mg/day), followed by outpatient oral therapy (trametinib 1 mg/0.5 mg/day alternating + dexamethasone 3.2 mg/day).

After four chemotherapy cycles, BCVA improved to 20/320 (right eye) and remained LP (left eye). Fundus exam showed reduced choroidal lesions, resolved SRF, retinal reattachment and scattered retinal pigment deposits in the right eye; left eye had partial lesion regression, perilesional retinal pigment deposit, posterior pole subretinal fibrosis, and attenuated/obliterated retinal arteries (Figures 1G,H). SS-OCT confirmed complete SRF resolution and retinal reattachment in the right eye, with residual hyperreflective subretinal material over lesions and marked improvement of choroidal lesions (Figures 2G–I). Pre- and post-treatment MRI revealed complete resolution of abnormal signals in the right eye and marked reduction in the left eye. The largest intracranial lesion achieved 78% volumetric regression.

Multidisciplinary review confirmed ECD with intraocular involvement. The patient continues scheduled chemotherapy and undergoes regular ophthalmic monitoring.

## 3 Discussion

ECD predominantly affects adults, with fewer than 20 pediatric cases reported to date (3). Given that systemic histiocytic disorders in children are commonly attributed to Langerhans cell histiocytosis (LCH), ECD is often not considered during the initial clinical, radiological, and histopathological evaluation (9). Furthermore, a comprehensive clinical and molecular characterization of pediatric ECD remains lacking. Consequently, the diagnosis of pediatric ECD may be significantly delayed, often for several years. In our case, although the two pathological findings are inconsistent, there are literature reports the coexistence of immunohistochemical markers of multiple histiocytic disorders occurring in the same patient, or even within the same biopsy specimen. This phenomenon is particularly common in ECD and LCH, especially in pediatric populations (10). Theoretically, an abnormality in a common progenitor cell (CD34+) or a shared inciting factor may underlie the coexistence of the two disease entities (11).

Due to the extreme rarity of intraocular involvement in ECD, the diagnosis may be missed, and may be misdiagnosed as a Choroidal melanoma. Choroidal melanoma typically presents as a unilateral lesion, and grows more slowly, forming mushroom-shaped choroidal masses that typically breach Bruch’s membrane, with dense cellular clusters that exhibit low to medium homogeneous echogenicity and a dome-shaped tumor with fluffy photoreceptor layers on OCT. The patient in this case report was asymptomatic prior to the incidental detection of bilateral choroidal masses. Based on multimodal fundus imaging, pathological biopsy, and significant improvement post-treatment, the findings are consistent with ECD involving the choroid.

Ocular involvement in ECD include palpebrae, orbit (12), ophthalmoplegia (13), cornea (14), anterior chamber, vitreous (7), and retina (15). Histiocytic choroidal infiltration presents as yellow or yellow-white lesions. According to literature reports (10), the median age at symptom onset and diagnosis is 5 and 6 years respectively, consistent with the case we report. In comparison to adult patients with ECD, the frequency of CNS and facial/orbital involvement is higher in pediatric cases, as indicated by our case (10).

In this case, the right eye demonstrated mild involvement, allowing for clearer observation of choroidal lesions. The primary manifestations included three orange-red, round choroidal infiltrative lesions in the right eye, accompanied by SRF. At the retinal level, SS-OCT showed HREM between the neurosensory retina and RPE, complete disruption of the ellipsoid zone corresponding to the choroidal lesions, shark-tooth-like alterations extending from the outer plexiform layer to the outer nuclear layer, and associated SRF surrounding the lesion periphery in the right eye. The progression of these lesions may result in pigmentary clumping and subretinal fibrosis. At the choroidal level, SS-OCT showed hyporeflective choroidal lesion, the obliteration of Haller’s layer, accompanied by subfoveal choroidal thickening, which aligned with existing reports (16).

HREM and SRF may be caused by a secondary inflammatory process owing to histiocytic infiltration of the choroid. The only reported choroidal biopsy demonstrated histiocytic infiltration within the choroid of ECD and supported the theory that the choroidal lesions with serous RD were most likely not secondary to a paraneoplastic inflammatory response (17). In certain adult patients, the ellipsoid zone corresponding to choroidal infiltration remained relatively preserved. In contrast, in our case, the ellipsoid zone was entirely disrupted. The underlying reasons for this discrepancy warrant further investigation through a larger number of clinical cases. Shark-tooth-like alterations have been described for the first time in cases of ECD, observed during periods of rapid disease progression, and may serve as a potential indicator of disease advancement. The thicker choroid of histiocytosis, a site of extramedullary hematopoiesis, may relate to the myeloid expansion that underlies the pathophysiology of the disease.

## 4 Conclusion

Overall, from an ophthalmological perspective, we have reported a pediatric ECD case involving choroidal and central nervous system, which contributes to addressing the current gap in the literature regarding intraocular manifestations of pediatric ECD.

## Data Availability

The raw data supporting the conclusions of this article will be made available by the authors, without undue reservation.
